# The Translation and Validation of the 18‐Item Davos Assessment of Cognitive Biases Scale in Arabic

**DOI:** 10.1002/brb3.71580

**Published:** 2026-07-09

**Authors:** Feten Fekih‐Romdhane, Nelly Kheir, Nour Zarrougui, Carlos Laranjeira, Farah Ghrissi, Majda Cheour, Sana Ellini, Souheil Hallit

**Affiliations:** ^1^ The Tunisian Center of Early Intervention in Psychosis, Department of Psychiatry “Ibn Omrane,” Razi Hospital Manouba Tunisia; ^2^ Faculty of Medicine of Tunis Tunis El Manar University Tunis Tunisia; ^3^ Faculty of Medicine Paris‐Saclay University Le Kremlin‐Bicêtre France; ^4^ School of Health Sciences, Campus 2 Polytechnic University of Leiria Leiria Portugal; ^5^ Centre For Innovative Care and Health Technology (ciTechCare) Polytechnic University of Leiria Leiria Portugal; ^6^ Comprehensive Health Research Centre (CHRC) University of Évora Évora Portugal; ^7^ School of Medicine and Medical Sciences Holy Spirit University of Kaslik Jounieh Lebanon; ^8^ Applied Science Research Center Applied Science Private University Amman Jordan

**Keywords:** Arabic, cognitive biases, DACOBS, psychometric properties, self‐report, validation

## Abstract

**Background:**

Validated assessment tools to measure cognitive biases in Arabic‐speaking contexts are still lacking, and this study proposes to fill this gap by translating and validating the 18‐item Davos Assessment of Cognitive Biases Scale (DACOBS) in Arabic. The specific objectives are the following: (1) examine the factor structure and internal consistency reliability of an Arabic translation of the DACOBS, (2) test measurement invariance across sex, and (3) investigate correlations with measures of psychotic experiences and delusional ideation.

**Method:**

Cross‐sectional data were collected online from Tunisian general population individuals over 18 years of age during the period from August 2025 to January 2026.

**Results:**

The 18 items of the scale loaded into four factors. The internal consistency of the Arabic DACOBS‐18 was acceptable to excellent, with a Cronbach's *α* value of 0.76 for the total scale and ranging from 0.69 to 0.80 for the four subscales. Invariance of the DACOBS‐18 was supported in our study, implying that the scale has comparable functioning across male and female samples. Our results showed a lack of sex differences in DACOBS‐18 mean scores. Finally, DACOBS‐18 total score and different sub‐scores correlated positively with self‐reported delusional ideation and positive psychotic experiences more broadly.

**Conclusion:**

Our study offers the first valid, reliable and practical instrument to assess cognitive biases in Arabic‐speaking contexts. Making available the DACOBS‐18 for self‐report use among Arabic‐speaking individuals could facilitate a more comprehensive evaluation of cognitive biases in local Arab contexts and enhance clinical practice in the field of psychosis in the region.

## 1 Introduction

Cognitive biases are defined as a metacognitive process that causes systematic errors in the elaboration of meaning across specific situations and times (Beck 1964). They are generally classified based on their content and cognitive processes underlying the elaboration of meaning: memory, reasoning, attributional, attentional, and motivational biases (Rector et al. 2011). Cognitive biases are hypothesized to play a crucial role in the etiology of psychosis. According to the cognitive model of psychosis (Garety et al. 2001; Moritz et al. 2017), biased information processing contributes to the development of psychotic symptoms in predisposed individuals. Empirical evidence supports that delusional experiences, both in clinical and non‐clinical populations, are closely related to proneness to jumping to conclusions (McLean et al. 2017; Ross et al. 2015) and attributional biases (So et al. 2015), inclination to discount contradictory evidence (Zawadzki et al. 2012), and a tendency to focus on threats (Gawęda et al. 2015). Furthermore, evidence has shown that cognitive biases allow to differentiate between patients with schizophrenia and healthy individuals (Riccaboni et al. 2012), and are present since the early stages of psychosis (e.g., in at‐risk state for psychosis [Peláez et al. 2026]), even in otherwise healthy individuals who experience subclinical positive psychotic symptoms (Livet et al. 2020). This scientific evidence suggests that cognitive biases may be promising targets for psychosis prevention and highlights the importance of their measurement using standardized assessment tools.

### 1.1 Measurements of Cognitive Biases

While several experimental tasks exist that can assess cognitive biases in psychosis (such as the Picture To Decision Task [Rubio et al. 2011] or the “beads” task [Speechley et al. 2010]), it has only been for a few years that self‐report instruments have been developed and validated as practical measures of cognitive biases for routine clinical use (such as the Cognitive Biases Questionnaire for Psychosis [CBQ‐P; [Peters et al. 2014] or the Davos Assessment of Cognitive Biases Scale [DACOBS; [van der Gaag et al. 2013]). The CBQ‐P was designed to assess five cognitive distortions relevant to psychosis and, more specifically, delusions: Dichotomous Thinking (i.e., “black or white”), Emotion‐Based Reasoning, Catastrophizing (i.e., worst‐case‐scenario thinking), Intentionalizing (i.e., interpreting events or behaviors as deliberate), and Jumping‐To‐Conclusions (i.e., making firm decisions based on little evidence). However, later empirical findings showed high correlations between the factors suggesting that the different cognitive biases assessed cannot be differentiated using the CBQ‐P. In addition, the CBQ‐P showed no correlations with experimental tasks (such as the Beads Task) and other self‐report measures of reasoning biases, suggesting that the scale does not cover judgment, reasoning, or decision‐making processes but should rather be regarded as a unidimensional measure of bias of interpretation (Peters et al. 2014). In contrast, the DACOBS subscales showed significant and adequate patterns of correlations with other relevant measures of common thinking biases, including Belief Inflexibility bias, attention to threat bias, and jumping to conclusions bias (van der Gaag et al. 2013).

### 1.2 The Original 42‐Item DACOBS

The DACOBS is intended to capture cognitive biases specific to positive symptoms of psychosis. It was originally conceptualized as a multidimensional tool composed of 42 items and seven dimensions: (1) external attribution biases, (2) attention to threat bias, (3) belief inflexibility bias, (4) jumping to conclusions bias, (5) subjective cognitive problems, (6) social cognition problems, and (7) safety behaviors. The first four subscales assess four cognitive biases, and the next two subscales measure cognitive limitations, while the last subscale evaluates safety behaviors (i.e., behavioral changes in response to a presumed threat). The scale was initially tested and validated in English in samples from the Netherlands. It showed satisfactory reliability (Cronbach's *α* ranging from 0.64 to 0.90) and was able to adequately distinguish patients with schizophrenia spectrum disorders from healthy controls (van der Gaag et al. 2013). The 42‐item DACOBS has been widely used, and its psychometric properties have been verified and established in various samples, including Flemish (Bastiaens et al. 2013), Italian (Pugliese et al. 2022), Turkish (Korkmaz et al. 2024), and Spanish (Pena‐Garijo et al. 2022) populations.

### 1.3 The Short 18‐Item Version of the DACOBS

A shorter version made up of 18 items was developed later and validated in the Polish language, with a four‐factor structure exhibiting an optimal model fit: (1) external attribution, (2) social cognition, (3) subjective cognition problems, and (4) safety behaviors (Gawęda et al. 2018). The 18‐item version showed good internal consistency and test–retest reliability, adequate discriminative ability, and significant positive correlations with psychotic symptoms and experiences. Other subsequent studies confirmed the good psychometric properties of the short version of the DACOBS in other languages and countries, including a Persian version in Iran (Aminaee et al. 2025) and a French version in Canada (Livet et al. 2023). These versions supported the four‐factor solution with 18 items, with satisfactory reliability and validity. The Persian version showed significant correlations with positive psychotic symptoms, paranoid ideation, schizotypal personality, and social cognition in clinical (i.e., patients with schizophrenia and major depressive disorder) and non‐clinical groups (Aminaee et al. 2025). The French version showed significant correlations with psychotic experiences in a sample of high schools and undergraduate students (Livet et al. 2023).

### 1.4 The Arab Context and the Present Study

Yet, the DACOBS‐18 has not been validated in the Arabic language, and its applicability and suitability in Arab cultural backgrounds remain a question. There are cultural variations in cognition expression, and research suggest that cognitive biases can be subject to cultural differences and might not be as universal as thought (Moser et al. 2022). Tunisia is a North‐African, Arab country typically classified as a collectivist society, which means that Tunisians ascribe great importance to maintaining harmonious relationships with others in their primary group, base their “self” on others’ views, and are generally concerned with being consistent with other members of the group (Hofstede 2011). Because the DACOBS‐18 was developed and validated within an individualistic Western context, an evaluation of its psychometric properties in a different cultural setting, such as the Tunisian community, is needed to determine whether the factor structure model of the scale holds up cross‐culturally. In addition, measurement invariance of the DACOBS‐18 across sex groups has been scarcely investigated so far, and the question whether there are sex‐dependent effects has yet to be examined.

In sum, validated assessment tools to measure cognitive biases in Arabic‐speaking contexts are still lacking, and this study proposes to fill this gap by translating and validating the 18‐item DACOBS in Arabic. The specific objectives are the following: (1) examine the factor structure and internal consistency reliability of an Arabic translation of the DACOBS‐18, (2) test measurement invariance of the scale across sex groups, and (3) investigate correlations with measures of positive psychotic experiences and delusional ideation.

## 2 Methods

### 2.1 Design, Sample, and Procedure

Cross‐sectional data were collected from Tunisian general population individuals over 18 years of age. Other eligibility criteria include having no personal history of previously diagnosed psychotic disorders or of antipsychotic intake, being able to read and understand written Arabic, and having access to the Internet. The study was conducted during the period from August 2025 to January 2026. An online questionnaire was created using Google Forms and distributed to participants on various social media platforms. It took about 15 min on average to fill out. Sampling was performed using a combination of convenience and snowball sampling techniques.

### 2.2 Ethical Considerations

The study's protocol was approved by the ethics committee of the Razi psychiatric hospital, Manouba, Tunisia. Participation was on a voluntary basis. Before filling the survey, each participant was asked to provide online consent after reading information about the study and its purposes. There was no compensation offered for participation in this study.

### 2.3 Minimal Sample Size Estimation

A minimal sample size of 360 participants was calculated, based on the rule of 20 participants per item (Mundfrom et al. 2005). This criterion was met with a final sample in the current study of 706 participants.

### 2.4 Measurements

#### 2.4.1 The 18‐Item DACOBS

This is a reduced version of the original 42‐item DACOBS (van der Gaag et al. 2013). It encompasses four dimensions reflecting self‐reported cognitive biases over the last 2 weeks (Gawęda et al. 2018). Response options vary on a 7‐point Likert‐type scale from 1 (*strongly disagree*) to 7 (*strongly agree*). The creation of an Arabic‐language version of the scale followed a number of steps based on international standards (Van Widenfelt et al. 2005). The creation of an Arabic‐language version of the scale followed a number of steps based on international standards (Van Widenfelt et al. 2005). The initial step was a forward‐backward translation process. Forward translation from English to the Modern Standard Arabic (MSA) was accomplished by a bilingual, native Arabic‐speaker health professional from Tunisia who was not involved in the research. This step ensured that the provisional Arabic version produced covered both the terminology used in the field of health and the language usually spoken in the MSA, while taking into consideration the Arab culture. Then, the back‐translation (from Arabic to English) was conducted by an English‐fluent psychiatrist from Tunisia who had sufficient knowledge about the culture‐related belief systems and who had not accessed the original DACOBS‐18. During these steps, the translators were instructed to ensure simplicity and clarity of the scale while adequately preserving items’ intended meanings and balancing literal and contextual translation. The next step consisted of comparing the two English versions (the original and the back‐translated) by a committee of experts who ensured that points of divergence are discussed and adequately resolved (Fenn et al. 2020). The committee of experts was composed of the two translators, a methodological expert, a psychiatrist, a psychologist, and the research team. Particular attention was given to evaluating item clarity, relevance, cultural appropriateness, and representativeness in order to guarantee semantic and conceptual equivalence of items, as well as the usability and applicability of the Arabic DACOBS‐18. An example of discrepancies that has been discussed is the translation of the term “task” (item 13) to Arabic, which was challenging as multiple translations (e.g., مهمة، واجب، عمل) convey distinct nuances related to duty, work, or activity. To preserve the intended cognition‐related construct and preserve the idea of sustained attention toward a specific activity, the preferred choice was “مهمة” because it better reflects a goal‐directed cognitive task. Another example is the English phrase “considering information” (item 18), for which Arabic provides several possible translations, each carrying distinct nuances that reflect distinct cognitive processes (e.g., thinking about = التفكير في المعلومات, evaluating = تقييم المعلومات, taking into account = أخذ المعلومات بعين الاعتبار, or examining information = النظر في المعلومات). To identify the wording that best captures the intended conceptual meaning, cognitive debriefing was used. To enhance comprehension and reduce interpretive bias, the translation that emphasizes taking information into account when making decisions or judgments (i.e., أخذ المعلومات بعين الاعتبار) was chosen. After minor linguistic adjustments to the preliminary translated version of the DACOBS‐18 into Arabic, a consensus was reached that the last version obtained was the best representation of the meaning of the items in Arabic. The last step was a pilot testing of the preliminary version using a sample of 30 general population adults to confirm that the Arabic items are clear and correctly understood. After this phase, no changes were deemed necessary.

#### 2.4.2 The Prodromal Questionnaire‐Brief (PQ‐B)

The Arabic version of the PQ‐B scale (Fekih‐Romdhane et al. 2024) was self‐administered to our general population participants to assess the frequency of positive psychotic experiences. This scale contains a total of 21 dichotomous (Yes/No) items (Loewy et al. 2011). Each “Yes” answer is scored with 1, while each “No” answer is scored with 0. Higher scores indicate greater psychotic experiences. In the current study, Cronbach's *α* for the total PQ‐B score was 0.92.

#### 2.4.3 The 21‐Item Peters et al. Delusions Inventory (PDI‐21)

The PDI‐21 was used to measure the severity of self‐reported delusional ideation in our non‐clinical sample. It is composed of 21 dichotomous (Yes/No) self‐rated items (Peters et al. 2004). Respondents who answer “yes” to an item are then asked to rate on a 5‐point Likert scale (1–5) levels of preoccupation, distress, and the degree of conviction associated with the delusional belief. Four independent scores can be obtained: number of beliefs, a conviction score, a preoccupation score, and a distress score. Only the first score was considered in the present study, which yielded a Cronbach's *α* of 0.82.

#### 2.4.4 Sociodemographic Information

Participants were asked to provide information about their age, sex, substance use (tobacco and alcohol), marital status, and any personal history of mental illness other than psychotic disorders that has been diagnosed by a primary care physician or a psychiatrist.

### 2.5 Statistical Analysis

Data analyses were conducted using the R software (Jorgensen et al. 2026; Rosseel et al. 2026). Multivariate normality was assessed using Mardia's test, which indicated significant multivariate skewness ( = 1665.55; *p* < 0.001) and kurtosis ( = 48.96; *p* < 0.001), suggesting violation of multivariate normality. Because DACOBS‐18 items are ordinal and exhibited non‐normal distributions, confirmatory factor analysis (CFA) was performed using the weighted least squares mean‐ and variance‐adjusted (WLSMV) estimator (Li 2016). Model fit was evaluated using multiple indices, including the comparative fit index (CFI) and Tucker–Lewis index (TLI), with values ≥ 0.90 indicating excellent fit, and the root mean square error of approximation (RMSEA) and standardized root mean square residual (SRMR), with values ≤ 0.08 considered acceptable (Byrne 2013). Internal consistency was assessed using McDonald's omega coefficient. Convergent validity was evaluated by calculating the average variance extracted (AVE). Measurement invariance across sex was examined sequentially at the configural, metric, scalar, and strict levels by comparing changes in CFI, RMSEA, and SRMR with established cut‐offs used to determine invariance (Chen 2007). Group differences in DACOBS‐18 scores were examined using the independent samples *t* test following the normal score distribution as verified by skewness and kurtosis values varying between −1 and +1 (Hair et al. 2021). Concurrent validity was assessed using Pearson correlation coefficients. Statistical significance was set at *p* < 0.05.

## 3 Results

The final sample included in the analyses was 706 Tunisian, Arabic‐speaking general population adults. The majority were females (*n* = 501, 71.0%). The mean age of the participants was 25.31 ± 4.08 years. Additional participants’ characteristics are presented in Table 1.

**TABLE 1:** Sample demographic and other characteristics (*N* = 706).

Variable	Mean ± SD or N (%)
DACOBS‐18 total score	52.20 ± 18.90
DACOBS‐18—Subjective cognitive problem	15.60 ± 6.68
DACOBS‐18—Safety behaviors	11.60 ± 5.40
DACOBS‐18—Attributional biases	11.40 ± 5.00
DACOBS‐18—Social cognitive problems	13.70 ± 5.35
**Marital status**	
Single, divorced, or widowed	654 (92.6%)
Married	52 (7.4%)
**Personal history of mental illness other than psychotic disorders**	
No	429 (60.8%)
Yes	277 (39.2%)
**Tobacco use**	
No	505 (71.5%)
Yes	201 (28.5%)
**Alcohol consumption**	
No	507 (71.8%)
Yes	199 (28.2%)

### 3.1 CFA

A four‐factor CFA was conducted to examine the factorial validity of the DACOBS‐18 using the WLSMV estimator for ordinal data. The model demonstrated good overall fit to the data (CFI = 0.989, TLI = 0.987, SRMR = 0.053, RMSEA = 0.061, 90% CI = 0.055–0.067). The model yielded *χ* ^2^ (129) = 470.55, *p* < 0.001.

**FIGURE 1:**
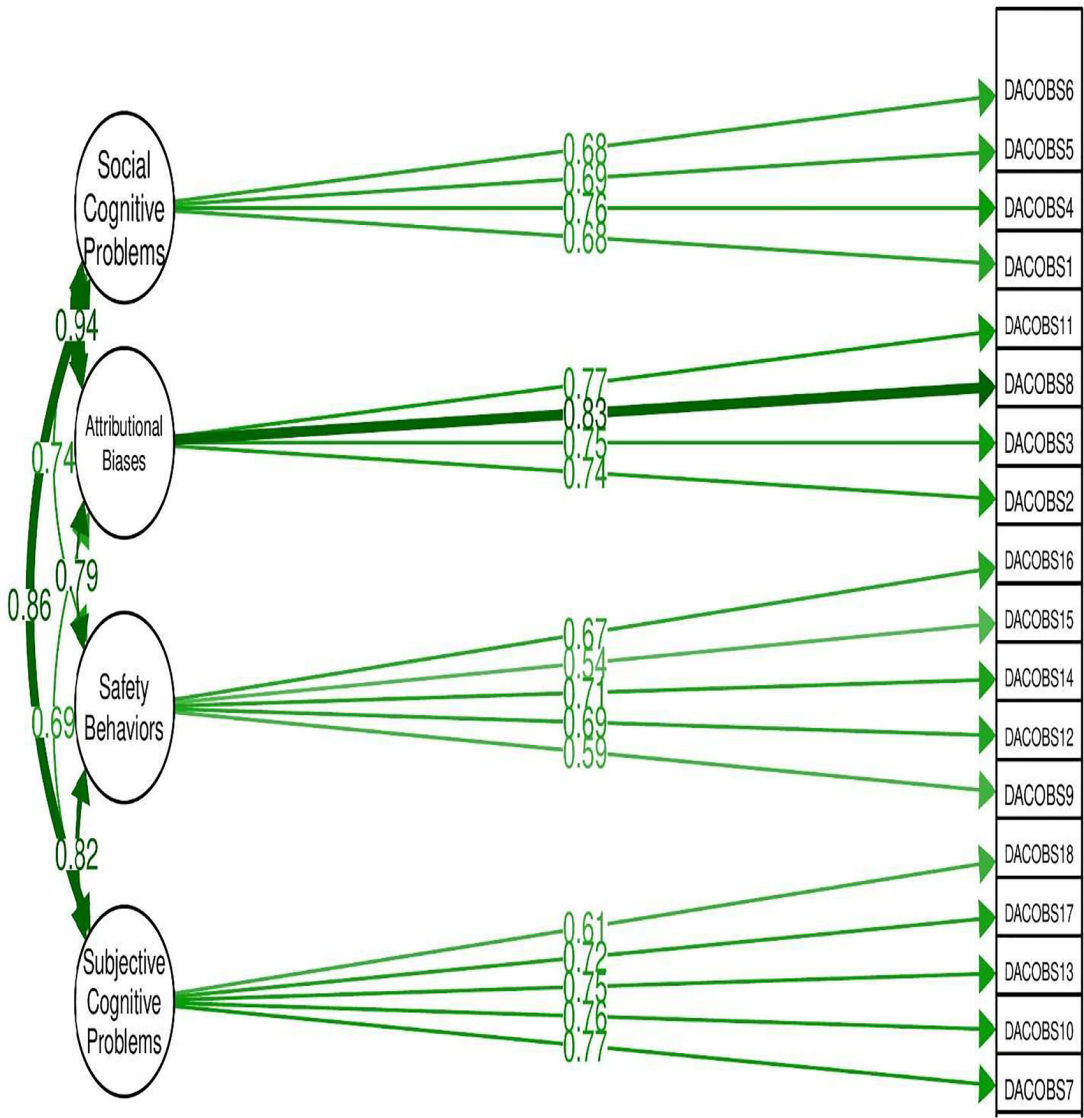
Confirmatory factor analysis of the DACOBS‐18. Standardized factor loadings are shown. All loadings were statistically significant (*p* < 0.001).

All standardized factor loadings were statistically significant (*p* < 0.001), with values ranging from 0.544 to 0.829 (Figure 1; Table 2). The proportion of explained variance (*R* ^2^) ranged from 0.295 to 0.687. Latent factor correlations were high (up to approximately 0.94), suggesting substantial overlap between dimensions and limited discriminant validity among some subscales. Therefore, although the theorized four‐factor structure demonstrated acceptable fit and all factor loadings were statistically significant, the findings should be interpreted as reflecting related and partially overlapping cognitive bias domains rather than fully independent constructs. These findings support the original four‐factor structure of the DACOBS‐18 in this sample, although the high correlations observed between latent factors suggest substantial overlap between dimensions.

**TABLE 2:** Item descriptive statistics and standardized factor loadings for the DACOBS‐18 (*N* = 706).

Factor	Item	Mean	SD	Standardized loading (*λ*)	*R* ^2^
1. **Subjective cognitive problems**	**DACOBS 7**. When I try to concentrate on something, it's hard to ignore other things around me.	4.00	1.86	0.771	0.594
	**DACOBS 10**. I get easily distracted by irrelevant information.	3.59	1.95	0.757	0.573
	**DACOBS 13**. I'm not able to focus on a task.	2.85	1.85	0.745	0.555
	**DACOBS 17**. It's hard to hold onto a thought.	2.81	1.73	0.716	0.513
	**DACOBS 18**. I avoid considering information which will disconfirm my beliefs.	2.34	1.61	0.608	0.369
2. **Safety behaviors**	**DACOBS 9**. I don't go out after dark.	2.32	1.72	0.587	0.344
	**DACOBS 12**. I always sit near the exit to be safe.	2.06	1.53	0.690	0.476
	**DACOBS 14**. People I don't know are dangerous.	2.42	1.58	0.710	0.504
	**DACOBS 15**. There is usually only one explanation for a single event.	2.09	1.38	0.544	0.295
	**DACOBS 16**. I don't answer phone calls to be on the safe side.	2.69	1.80	0.674	0.454
3. **Attributional biases**	**DACOBS 2**. People cannot be trusted.	3.85	1.66	0.741	0.549
	**DACOBS 3**. Things went wrong in my life because of other people.	3.27	1.77	0.751	0.565
	**DACOBS 8**. People make my life miserable.	2.28	1.54	0.829	0.687
	**DACOBS 11**. People treat me badly for no reason.	1.96	1.33	0.766	0.587
4. **Social cognitive problems**	**DACOBS 1**. People confuse me.	3.22	1.70	0.676	0.457
	**DACOBS 4**. I am often not sure what people mean.	3.59	1.73	0.760	0.577
	**DACOBS 5**. When I have a goal, I do not know how to reach it.	3.32	1.81	0.690	0.476
	**DACOBS 6**. I do not understand why people react in a certain way.	3.53	1.77	0.677	0.458

*Note*. All factor loadings were statistically significant (*p* < 0.001).

Latent factor correlations were high (up to ≈ 0.94), suggesting substantial overlap between dimensions; however, the theorized 4‐factor structure showed adequate fit and all loadings were significant.

### 3.2 Second‐Order Model

A second‐order model including a higher‐order latent factor (Cognitive Bias) loading onto the four first‐order factors was also tested. The model demonstrated acceptable fit (CFI = 0.985, TLI = 0.982, SRMR = 0.059, RMSEA = 0.070, 90% CI = 0.065–0.076). The model yielded *χ* ^2^ (131) = 589.82, *p* < 0.001.

Although slightly inferior to the first‐order model, the second‐order solution remained within acceptable fit thresholds, supporting the conceptualization of a broader general cognitive bias construct underlying the four related dimensions. Taken together, the findings suggest that the DACOBS‐18 may capture both differentiated cognitive bias domains and a substantial higher‐order common cognitive bias factor.

### 3.3 Internal Consistency and Convergent Validity

Internal consistency was acceptable to excellent across subscales: subjective cognitive problems (*α* = 0.80; *ω* = 0.80), Safety Behaviors (*α* = 0.69; *ω* = 0.70), Attributional Biases (*α* = 0.80; *ω* = 0.80), Social Cognitive Problems (*α* = 0.76; *ω* = 0.76), and for total scale *α* = 0.76. Composite reliability values ranged from 0.778 to 0.855, indicating satisfactory reliability across subscales. The AVE values were as follows: Subjective Cognitive Problems = 0.521, Safety Behaviors = 0.415, Attributional Biases = 0.597, and Social Cognitive Problems = 0.492. AVE values exceeded the conventional 0.50 threshold for the Subjective Cognitive Problems and Attributional Biases factors, whereas the Safety Behaviors and Social Cognitive Problems factors showed AVE values slightly below 0.50, indicating moderate convergent validity and supporting a cautious interpretation of the distinctiveness of these dimensions.

### 3.4 Sex Invariance

Measurement invariance across sex was examined using multi‐group CFA with WLSMV estimator. The configural model demonstrated good fit (CFI = 0.990; RMSEA = 0.060; SRMR = 0.059), indicating that the four‐factor structure was comparable across male and female participants. Metric invariance was supported, as constraining factor loadings resulted in negligible changes in model fit (ΔCFI = −0.003; ΔRMSEA = 0.004; ΔSRMR = 0.004). Scalar and strict invariance were also supported, as further equality constraints on thresholds and residual variances did not meaningfully deteriorate model fit (ΔCFI ≤ 0.003). These results indicate full measurement invariance across sex (Table 3).

**TABLE 3:** Cross‐sex measurement invariance.

Model	CFI	RMSEA	SRMR	Model Comparison	ΔCFI	ΔRMSEA	ΔSRMR
Configural	0.990	0.060	0.059				
Metric	0.987	0.064	0.063	Configural vs. metric	−0.003	0.004	0.004
Scalar	0.990	0.051	0.059	Metric vs. scalar	0.003	−0.013	−0.004
Strict	0.989	0.053	0.063	Scalar vs. strict	−0.001	0.002	0.004

*Note*: Invariance was supported based on ΔCFI ≤ 0.010, ΔRMSEA ≤ 0.015, and ΔSRMR ≤ 0.010.

Abbreviations: CFI, comparative fit index; RMSEA, root mean square error of approximation; SRMR, standardized root mean square residual.

Following invariance confirmation, differences in DACOBS‐18 total scores between male and female participants were examined using an independent samples Welch *t*‐test. No statistically significant difference was found between sexes (50.69 ± 17.90 vs. 52.80 ± 19.30), *t* (407.36) = −1.39, *p* = 0.165. The effect size was small (Cohen's *d* = −0.11, 95% CI [−0.27, 0.05]), indicating comparable levels of cognitive biases across male and female participants.

### 3.5 Concurrent Validity

Higher DACOBS‐18 total scores were significantly and positively correlated with greater levels of delusional ideation (*r* = 0.47, *p* < 0.001) and psychosis‐risk experiences (*r* = 0.55, *p* < 0.001) (Table 4). Similar patterns were observed across the four DACOBS subscales.

**TABLE 4:** Correlation matrix between scores.

Variable	1	2	3	4	5	6	7
1. DACOBS‐18 Total score	1						
2. DACOBS‐18—Subjective Cognitive Problem	0.87***	1					
3. DACOBS‐18—Safety Behaviors	0.80***	0.61***	1				
4. DACOBS‐18—Attributional Biases	0.83***	0.55***	0.57***	1			
5. DACOBS‐18—Social Cognitive Problems	0.87***	0.68***	0.54***	0.72***	1		
6. Delusional ideation (PDI)	0.47***	0.35***	0.37***	0.47***	0.43***	1	
7. Psychotic experiences (PQ‐B)	0.52***	0.44***	0.42***	0.47***	0.45***	0.64***	1

*Note*: Numbers in the table refer to Pearson coefficients.

*p* < 0.05; *p* < 0.01; ***p < 0.001.

## 4 Discussion

Few studies have examined delusion‐associated cognitive biases in Arab populations. Therefore, there is a clear need to investigate the psychometric properties of the DACOBS‐18 in an Arabic‐speaking sample. The aim of our study was to validate the Arabic version of the DACOBS‐18. Findings showed that the 18 items of the scale loaded into four factors as expected and demonstrated acceptable internal consistency. The results also showed substantial evidence for measurement invariance across male and female participants, as well as significant correlations with delusional ideation and broader positive psychotic experiences.

Concerning the factor structure of the new Arabic scale, CFA supported a four‐factor model: a first factor consists of four items covering Attributional Biases, a second factor involves four items that assess Social Cognition Problems, a third factor has five items measuring the severity of Safety Behaviors associated with interpersonal threat, and a fourth factor is composed of five items related to Subjective Cognitive Problems. In agreement with our findings, the Polish version of the DACOBS‐18 was the first to describe a four‐factor model as the best fit for their data using a large sample of clinical and non‐clinical adults (Gawęda et al. 2018). Subsequently, a French version of the scale could replicate the four‐factor solution in 213 students from Canada (Livet et al. 2023), and a Persian version supported the same four‐dimensional structure using 18 items in patients with schizophrenia or major depressive disorder and healthy subjects (Aminaee et al. 2025). Therefore, the four underlying dimensions of the cognitive biases construct as assessed using the DACOBS‐18 appear to be consistent across different linguistic and cultural contexts. Although the four‐factor structure demonstrated acceptable fit and replicated the theoretical organization of the original DACOBS‐18, the relatively high correlations observed between latent factors suggest substantial conceptual overlap among the dimensions. This finding indicates that the proposed subscales may reflect closely related aspects of a broader cognitive bias construct rather than entirely distinct domains. The acceptable fit of the second‐order model further supports the presence of an overarching higher‐order cognitive bias factor underlying the four dimensions. Additionally, the AVE values below the conventional 0.50 threshold for the Safety Behaviors and Social Cognitive Problems factors suggest that convergent and discriminant validity should be interpreted cautiously. Future studies should continue examining the dimensional structure of the DACOBS‐18 across different populations and clinical samples.

Furthermore, the internal consistency reliability of the Arabic DACOBS‐18 was acceptable to excellent, with a Cronbach's *α* value of 0.76 for the total scale and ranging from 0.69 to 0.80 for the four subscales. Our results align with those of other studies. The Polish version yielded a Cronbach's *α* for the DACOBS‐18 total score of 0.84 (Gawęda et al. 2018). In the Canadian French‐speaking sample, Cronbach's *α* values varied between 0.62 (the safety behavior scale) and 0.81 (the external attribution biases), and was of 0.86 for the DACOBS total score (Livet et al. 2023). In the Iranian Persian‐speaking sample, Cronbach *α* varied between 0.70 (social cognition problems) and 0.71 (attributional biases) and were 0.86 for the DACOBS‐18 total score in the non‐clinical sample (Aminaee et al. 2025).

The question of measurement invariance of cognitive biases measures has rarely been examined in previous studies. Invariance of the DACOBS‐18 was supported in our study, implying that the scale has comparable functioning across male and female samples. This suggests that mean score comparisons across sex groups are valid and not affected by measurement biases, which is a prerequisite for drawing meaningful conclusions in this regard. Our results showed a lack of sex differences in DACOBS‐18 mean scores. This is consistent with other previous research findings that indicated no sex differences in jumping‐to‐conclusions and evidence integration impairment both in patients with schizophrenia spectrum disorders and healthy controls (de Vos et al. 2019). Likewise, another study reported no significant sex differences in cognitive biases (i.e., jumping‐to‐conclusions) in patients with recent‐onset psychotic disorders (Gonzalez et al. 2018).

Another objective of this study was to explore the concurrent validity of the Arabic DACOBS‐18 by examining the correlations between cognitive biases and theoretically relevant variables. Findings showed that DACOBS‐18 total score and different sub‐scores correlated positively with self‐reported delusional ideation and positive psychotic experiences more broadly. These findings extend those from previous studies to a new geographical context (Tunisia and North Africa) and further highlight the consistent relationship between cognitive biases and positive psychotic symptoms (Moritz et al. 2017). In particular, the sub‐dimension of external attribution bias was the most strongly correlated with delusional ideation, followed by social cognition problems. Consistently, social cognition problems were found to play a crucial role in the early phases of psychosis, and external attribution bias was shown to contribute substantially to the emergence and persistence of positive psychotic symptoms (Jáni and Kašpárek 2018; Lee et al. 2015). Together, these observations indicate that self‐reported cognitive biases are clinically relevant, as they may reflect an increased risk for developing psychotic symptoms in healthy young people (Pot‐Kolder et al. 2018).

### 4.1 Limitations

Findings need to be interpreted in the context of some limitations. First, our study relied exclusively on self‐reported measures, which are subject to many biases such as social desirability and the risk of misunderstanding or hiding. The second limit of the study is the adoption of an online survey as the data collection method, which can reduce the generalizability of the study results, as Internet users might not fully represent the general population. Furthermore, all validity evidence in the study was derived from self‐report measures, which introduces the possibility of mono‐method bias. In addition, a notable limitation of the present study is that test–retest reliability was not investigated. Consequently, the temporal stability of the instrument over time could not be determined. This important psychometric property remains to be verified in future research. Finally, only a non‐clinical population was involved, which precluded us from examining discriminant validity of the Arabic DACOBS‐18. To address this limitation, future studies need to include both patients with psychotic disorders and healthy participants and investigate the extent to which the Arabic DACOBS‐18 is able to differentiate people diagnosed with psychosis from controls.

### 4.2 Implications

This study presents preliminary psychometric evidence from a Tunisian Arabic‐speaking community sample that the 18‐item Arabic DACOBS is valid and reliable for measuring self‐report cognitive biases as a multidimensional construct in community Arabic‐speaking individuals. Making available the DACOBS for self‐report use among Arabic‐speaking individuals could facilitate a more comprehensive evaluation of cognitive biases in local Arab contexts and enhance clinical practice in the field of psychosis (and young people's mental health more broadly) in the region. Given its ease of use, the Arabic DACOBS‐18 can help inform decision‐making in clinical settings by aiding in the identification of individuals at risk for psychosis. However, this can only be possible after extending the present findings to clinical samples to establish the discriminant validity of the scale. Finally, the new Arabic scale will hopefully offer significant potential to advance future research and contribute to improved comparability of international findings.

## 5 Conclusion

To summarize, our findings lend further support to those of previous psychometric studies, showing that the 18‐item DACOBS is a psychometrically sound measure to assess general thinking bias related to psychotic phenomena within a Tunisian, North‐African population. Our study offers a preliminary valid, reliable and practical instrument to assess cognitive biases in Arabic‐speaking contexts. We hope that the Arabic DACOBS‐18 can be integrated in clinical practice to promote early recognition and ensure timely referral for care. It is also hoped that the Arabic DACOBS will provide new insights to shape and guide future research in the field in the Arab region and abroad.

## Author Contributions

**Feten Fekih‐Romdhane**: conceptualization, investigation, writing – original draft, writing – review and editing, visualization, validation, methodology, supervision. **Nelly Kheir**: formal analysis, writing – review and editing. **Nour Zarrougui**: data curation, writing – review and editing. **Carlos Laranjeira**: writing – review and editing, visualization, validation. **Farah Ghrissi**: writing – review and editing, validation, visualization. **Majda Cheour**: writing – review and editing, visualization, validation. **Sana Ellini**: writing – review and editing, visualization, validation. **Souheil Hallit**: software, formal analysis, methodology, writing – review and editing.

## Funding

The authors have nothing to report.

## Ethics Statement

Each participant provided a voluntary, online informed consent before beginning the survey. The research protocol was approved by the ethics committee of the Razi psychiatric hospital, Manouba, Tunisia. The study was performed following the standards for medical research involving human subjects recommended by the Declaration of Helsinki for human research.

## Consent

The authors have nothing to report.

## Conflicts of Interest

The authors declare no conflicts of interest.

## Data Availability

The datasets generated and/or analyzed during the current study are not publicly available due to restrictions from the ethics committee but are available from the corresponding author (S.H.) on reasonable request.
